# Stone heterogeneity index on single-energy noncontrast computed tomography can be a positive predictor of urinary stone composition

**DOI:** 10.1371/journal.pone.0193945

**Published:** 2018-04-12

**Authors:** Jong Soo Lee, Kang Su Cho, Seung Hwan Lee, Young Eun Yoon, Dong Hyuk Kang, Won Sik Jeong, Hae Do Jung, Jong Kyou Kwon, Joo Yong Lee

**Affiliations:** 1 Department of Urology, Severance Hospital, Urological Science Institute, Yonsei University College of Medicine, Seoul, Korea; 2 Department of Urology, Gangnam Severance Hospital, Urological Science Institute, Yonsei University College of Medicine, Seoul, Korea; 3 Department of Urology, Hanyang University College of Medicine, Seoul, Korea; 4 Department of Urology, Inha University School of Medicine, Incheon, Korea; 5 Department of Urology, Kwangju Christian Hospital, Gwangju, Korea; 6 Department of Urology, Yongin Severance Hospital, Yonsei University Health System, Yongin, Korea; 7 Department of Urology, Severance Check-Up, Yonsei University Health System, Seoul, Korea; University of Oxford, UNITED KINGDOM

## Abstract

The aim of this study was to investigate the correlation between stone composition and single-energy noncontrast computed tomography (NCCT) parameters, including stone heterogeneity index (SHI) and mean stone density (MSD), in patients with urinary calculi. We retrospectively reviewed medical records of 255 patients who underwent operations or procedures for urinary stones or had spontaneous stone passage between December 2014 and October 2015. Among these, 214 patients with urinary calculi who underwent NCCT and stone composition analyses were included in the study. Maximal stone length (MSL), mean stone density (MSD), and stone heterogeneity index (SHI) were determined on pretreatment NCCT. The mean MSD (454.68±177.80 HU) and SHI (115.82±96.31 HU) of uric acid stones were lower than those of all other types. Based on post hoc tests, MSD was lower for uric acid stones than for the other types (vs. CaOx: P<0.001; vs. infection stones: P<0.001). SHI was lower for uric acid stones than for the other types (vs. CaOx: P<0.001; vs. infection stones: P<0.001) Receiver operating characteristic curves of uric acid stones for MSD and SHI demonstrated that SHI (cut-off value: 140.4 HU) was superior to MSD (cut-off value: 572.3 HU) in predicting uric acid stones (P<0.001).

## Introduction

Pretreatment determination of urinary stone composition can be essential for optimal stone management [1]. It is important for three reasons. Firstly, the composition is related to hardness, which in turn affects the outcomes of extracorporeal shockwave lithotripsy (ESWL), ureteroscopic lithotripsy (URSL), and retrograde intra-renal surgery (RIRS) [2]; hard stones may be resistant to shock-wave and Holmium:YAG laser [3]. Mean stone density (MSD), as represented by the mean Hounsfield unit (HU) value measured during non-contrast computed tomography (NCCT), can be related to stone hardness [4]. Secondly, stones occurring with various metabolic syndromes, such as cysteine stones or uric acid stones, may require systemic medical treatment for chemolitholysis [5]. Finally, knowing a stone’s composition is useful for preventive efforts, and it can be a condition for chemoprevention and regulating life-style modification. [6].

Although stone composition is important for treatment decisions, it is often difficult to be certain of the composition. NCCT has become the gold standard for diagnosis of urolithiasis nowadays, as it provides information regarding stone size and location [7]. Furthermore, several studies have reported being able to predict stone characteristics with HUs, including several HU-related parameters [8].

Recently, the newly reported stone heterogeneity index (SHI), which is a proxy of stone variation, was defined as the standard deviation of the mean HU in a region of interest on NCCT; it was proposed as a novel predictor for ESWL outcomes in patients with ureteral stones [9]. Lee et al. reported that SHI could be a useful clinical parameter for stone fragility and hardness (which can affect the outcomes of ESWL) and an independent predictor of ESWL success rate. However, stone composition could not be analyzed in that study because of the effects of ESWL; this was a major limitation of their study. Thus, in the current study we investigated the correlation between pretreatment NCCT parameters, such as SHI and MSD, and stone composition.

## Materials and methods

### Patient cohort

Medical records were obtained from our hospital database for 255 patients who underwent surgical operations or procedures or had spontaneous urinary stone passage between December 2014 and October 2015 at Severance Hospital, Seoul, Korea. Inclusion criteria were as follows: (1) NCCT performed before treatment or spontaneous stone passage; and (2) the target stone measured by NCCT was retrieved and its stone composition was analyzed. Patients with urinary tract congenital anomalies, single kidney, or preoperative receipt of stone-dissolving medication (including potassium citrate, tiopronin, and antibiotics) were excluded from the study. Surgical operations or procedures included URSL, ESWL, percutaneous nephrolithotomy (PCNL), RIRS, vesicolitholapaxy (VESL), laparoscopic ureterolithotomy (LAPU), and laparoscopic pyelolithotomy (LAPP). A total of 214 patients were included in the study.

### Good clinical practice protocols

The study was performed in accordance with all applicable laws and regulations, good clinical practices, and ethical principles described in the Declaration of Helsinki. The Institutional Review Board of the Severance Hospital approved the study protocol (Approval No. 4-2015-0947). The study was exempted from requiring written informed consent from the participants because of its retrospective design and because the patients’ records and information were anonymized and de-identified prior to analysis.

### Stone characteristics on non-contrast computed tomography

Stone characteristics included the location, size, MSD, and SHI. We used the GE Centricity system (GE Healthcare Bio-Sciences Corp., Piscataway, NJ, USA) to obtain measurements. The stone size was determined from the largest stone diameter on the axial or coronal plane of NCCT. HU was measured on the magnified, axial NCCT image from the point of the largest stone diameter, where the elliptical region of interest incorporated the largest cross-sectional area of the stone without including the adjacent soft tissue. MSD was defined as the mean value of HU in the region of interest, and SHI was defined as the standard deviation of HU in the same region of interest. In this study, HU was measured by two researchers (H.D.J and J.S.L), and if there were any significant differences, the third researcher (J.Y.L) performed measurement again and corrected it. We measured target stones in both pre-treatment NCCT and post-treatment NCCT. In bilateral cases, we obtain two stone composition results; in this case, we divided two samples in dataset.

### Stone composition analysis

Stone composition quantitative analysis was performed using Fourier-Transform Infrared Spectrometry (FT-IRS). The FT-IRS was carried out in Green Cross Laboratories, Yongin, Korea.

### Statistical analysis

Data are presented as mean±standard deviation, except where otherwise indicated. Statistical comparisons of continuous variables from patient demographic information were performed using either Student’s or Welch’s two-sample t-tests or Wilcoxon rank-sum test. In the subgroup analyses, one-way analysis of variance (ANOVA) was used. After ANOVA, Tukey–Kramer’s post hoc tests were used for comparisons between groups. Categorical variables were compared using Pearson's chi-squared test. Optimal cut-off values for significant values were identified from receiver operating characteristic (ROC) curves using Youden methodology. Statistical analyses were performed using R software (version 3.3.2, R Foundation for Statistical Computing, Vienna, Austria; http://www.r-project.org) and its OptimalCutpoints package for determining the optimal cut-off values.

## Results

### Demographic and stone characteristics of all patients

**Fig 1:**
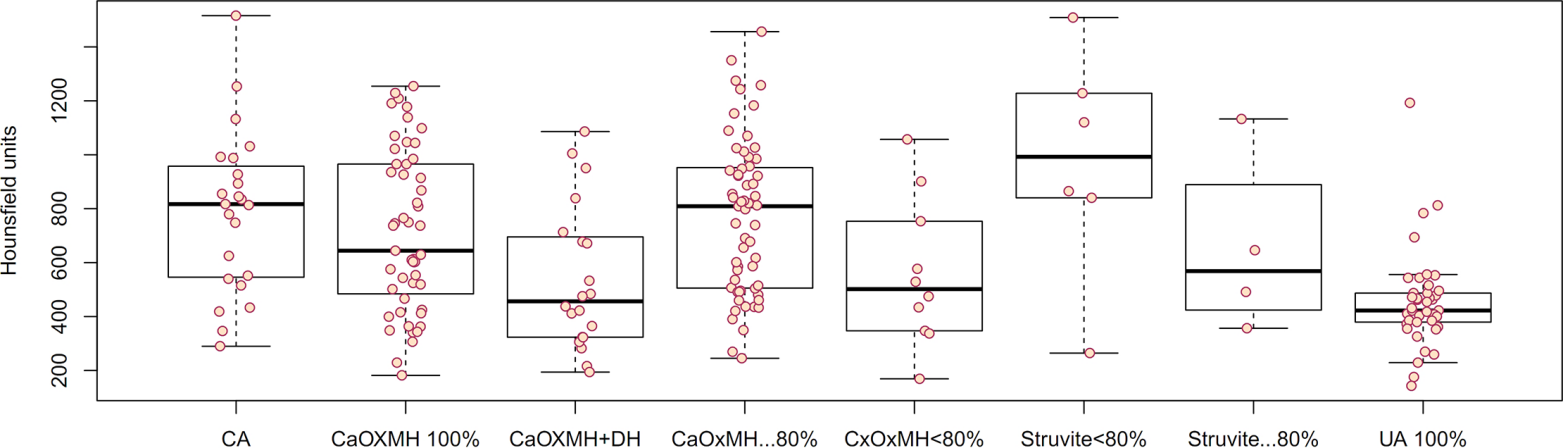
Mean stone density in noncontrast computed tomography values according to stone composition. CA: carbonate apatite; CaOx: calcium oxalate; MH: monohydrate; DH: dehydrate; UA: uric acid.

**Fig 2:**
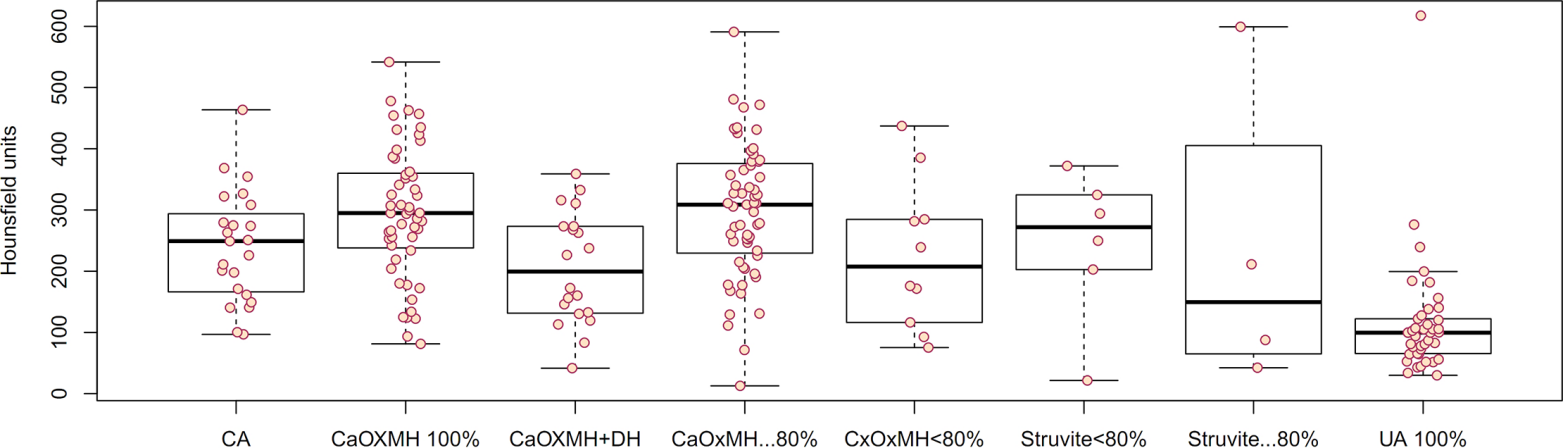
Stone heterogeneity index in noncontrast computed tomography values according to stone composition. CA: carbonate apatite; CaOx: calcium oxalate; MH: monohydrate; DH: dehydrate; UA: uric acid.

The mean age of total patients was 54.86±15.59 years. The distribution of operations and procedures included 9 cases of ESWL, 2 cases of LAPP, 14 cases of LAPU, 36 cases of PCNL, 19 cases of RIRS, 114 cases of URSL, and 12 cases of VESL. There were 8 cases of spontaneous stone passage. Sixty cases were renal stones. Ureter stones included 69 cases of upper ureter stones, 12 cases of midureter stones, and 62 cases of lower ureter stones. Eleven cases were bladder stones. The mean MSL was 12.77±9.07 mm, the MSD was 670.06±304.00 HU, and the SHI was 241.61±126.61 HU (Table 1). Stone composition analyses revealed 140 calcium oxalate (CaOx) stones, which included 51 monohydrate stones, 59 mixed stones with ≥ 80% CaOx monohydrate (MH), 10 mixed stones with < 80% CaOxMH, and 20 mixed CaOxMH and dehydrate (DH) stones. Infection stones included 23 carbonate apatite stones and 10 struvite stones. There were 41 cases of uric acid stones. Table 2 demonstrates the patient and stone characteristics according to stone composition. As shown in Figs 1 and 2, MSD (454.68±177.80 HU) and SHI (115.82±96.31) of uric acid stones were lower than for the other types of stones.

**Table 1:** Patient and stone data for the total cohort and three groups including calcium oxalate, infection, and uric acid stones.

	Total cohort	CaOx compound	Infection	Uric acid	P-value
**No. of patients**	214	140	33	41	
**Age**	54.86±15.59	52.56±16.19	55.67±13.81	62.05±12.61	0.002^a^
**Sex**					0.374^b^
**Male**		85	20	30	
**Female**		55	13	11	
**Procedures**					
**ESWL**	9	7	1	1	
**LAPP**	2	1	0	1	
**LAPU**	14	10	3	1	
**PCNL**	36	11	11	14	
**RIRS**	19	14	3	2	
**URSL**	114	90	10	14	
**VESL**	12	1	5	6	
**Spontaneous passage**	8	6	0	2	
**Location**					
**Kidney**	60	27	17	16	
**Upper ureter**	69	51	8	10	
**Midureter**	12	11	0	1	
**Lower ureter**	62	50	4	8	
**Bladder**	11	1	4	6	
**MSL**	12.77±9.07	9.61±5.05	19.61±12.49	18.05±11.08	<0.001^a^
**MSD**	670.06±304.00	701.09±297.37	806.01±329.87	454.68±177.80	<0.001^a^
**SHI**	241.61±126.61	278.73±112.10	240.43±119.28	115.82±96.31	<0.001^a^

Data are mean±standard deviation or number.

CaOx: calcium oxalate; ESWL: extracorporeal shock wave lithotripsy; LAPP: laparoscopic pyelolithotomy; LAPU: laparoscopic ureterolithotomy; PCNL: percutaneous nephrolithotripsy; RIRS: retrograde intra-renal surgery; URSL: ureteroscopic lithotripsy; VESL: vesicolitholapaxy; MSL: maximal stone length; MSD: mean stone density; SHI: stone heterogeneity index

a. Based on one-way ANOVA

b. Based on Pearson's chi-squared tests with Yates' continuity correction

**Table 2:** Patient and stone characteristics according to stone composition.

	n	Male:Female	Age (year)	Stone length (mm)	NCCT values (HU)			
					Min	Max	MSD	SHI
**CaOx**								
**MH 100%**	51	34:17	57.06±13.99	8.82±3.76	151.25±113.36	1187.90±379.33	716.06±297.52	295.27±107.86
**MH≥80%**	59	29:30	53.80±15.22	10.91±6.07	174.34±89.95	1248.81±402.86	768.47±287.83	298.14±109.92
**MH<80%**	10	6:4	53.10±14.42	10.04±4.44	146.70±133.89	937.80±417.17	557.91±273.49	225.91±122.36
**MH+DH**	20	16:4	37.15±16.98	7.58±4.00	175.05±81.46	889.55±380.79	535.72±267.20	205.69±91.36
**Carbonate apatite**	23	14:9	55.57±14.72	18.62±10.80	162.13±83.04	1182.78±396.46	788.90±299.48	240.42±92.05
**Struvite**								
**≥80%**	4	1:3	53.75±7.97	18.50±19.92	193.50±123.69	1067.50±764.64	656.62±338.68	235.08±253.03
**<80%**	6	3:3	57.33±14.94	24.17±14.75	306.50±130.58	1342.50±602.93	971.17±425.46	244.07±123.77
**Uric acid 100%**	41	11:30	62.05±12.61	18.05±11.08	167.83±97.02	676.93±348.89	454.68±177.80	115.82±96.31

Data are mean±standard deviation or number.

CaOx: calcium oxalate; MH: monohydrate; DH: dehydrate; NCCT: noncontrast computed tomography; Min: minimum; Max: maximum; MSD: mean stone density; SHI: stone heterogeneity index

### Analyses of three stone groups: Calcium oxalate, infection, and uric acid stones

As shown in Table 1, there were statistically significant differences for mean age, MSL, MSD, and SHI among the three groups of stones (CaOx compounds inclung MH and DH, infection stones, and uric acid stones). Based on post hoc tests, patients with CaOx were younger than those with uric acid stones (P = 0.005). The MSL was greater for uric acid stones than for the other types of stones (vs. CaOx: P<0.001; vs. infection stones: P<0.001); however, there were no differences in MSL between CaOx and infection stones (P = 0.679). The MSD was lower for uric acid stones than for the other types of stones (vs. CaOx: P<0.001; vs. infection stones: P<0.001). The SHI was lower for uric acid stones than for the other types (vs. CaOx: P<0.001; vs. infection stones: P<0.001); however, there were no significant differences in MSD (P = 0.139) and SHI (P = 0.175) between CaOx and infection stones.

### ROC curves and cut-off values of MSD and SHI for predicting uric acid stones

**Fig 3:**
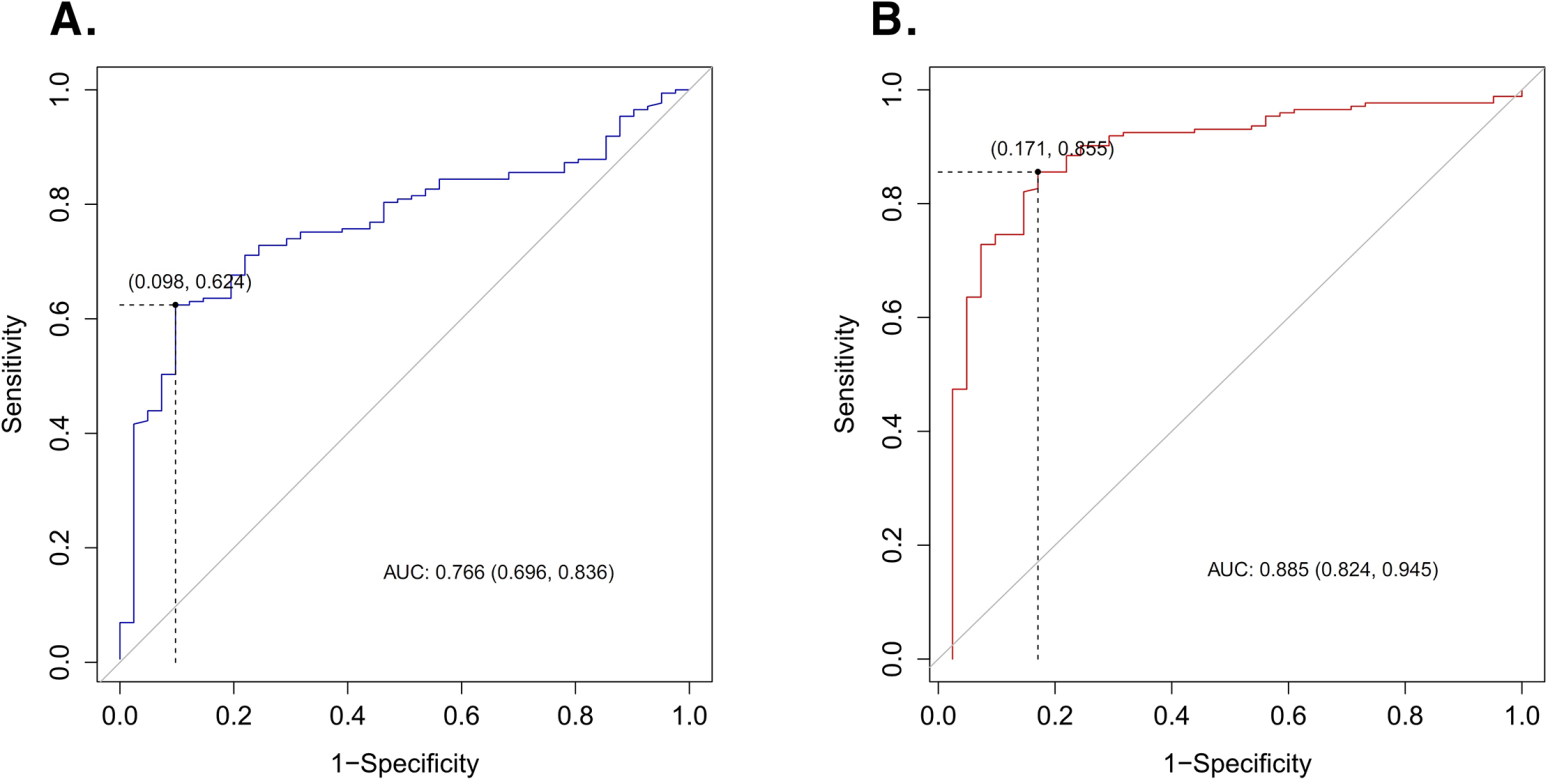
Receiver operating characteristic (ROC) curve of uric acid stones for mean stone density and stone heterogeneity index. (A) The area under the ROC curve (AUC) for the mean stone density was 0.766 (95% confidence interval [CI], 0.696–0.836) and the cut-off value was 572.3 HU. (B) The AUC for the stone heterogeneity index was 0.885 (95% CI, 0.824–0.945) and the cut-off value was 140.4 HU.

For MSD, the area under the ROC curve (AUC) was 0.766 (95% confidence interval [CI], 0.696–0.836), and the cut-off value was 572.3 HU. For SHI, the AUC was 0.885 (95% CI, 0.824–0.945), and the cut-off value was 140.4 HU (Fig 3). Comparing MSD to SHI in their ability to predict uric acid stones, SHI was superior to MSD based on DeLong’s test for two correlated ROC curves (P<0.001). Sensitivity and specificity of MSD for uric acid stones were 0.624 (95% CI 0.548–0.697) and 0.902 (95% CI 0.769–0.973), respectively. SHI demonstrated that sensitivity and specificity were 0.855 (95% CI 0.794–0.904) and 0.829 (95% CI 0.679–0.928) for uric acid stones. Positive predictive value was 0.964 (95% CI 0.907–0.974) and negative predictive value was 0.362 (95% CI 0.293–0.687) in uric acid stone using MSD. In SHI, positive and negative predictive values for uric acid stone were 0.955 (95% CI 0.902–0.971) and 0.576 (95% CI 0.470–0.784), respectively.

## Discussion

In the first study to examine the relationship between stone composition and NCCT parameters, Spettel et al. reported that uric acid stones exhibited significantly different MSD (as determined by the average HU on NCCT) compared with other stones [10]. They suggested that an MSD ≤ 500 and a pH ≤ 5.5 had a positive predictive value of 90% for uric acid composition in stones > 4 mm. Urine pH, the presence of crystals, urease-positive bacteria in the urine, plain x-ray appearance, and a history of urinary stones have long been used to predict the composition of stones [11]. However, a recent report showed no relationship between urine pH and stone composition. In their study evaluating the relationships among body mass index (BMI), visceral fat, urine pH, and stone composition, Kim et al. concluded that visceral fat adiposity strongly correlated with the presence of uric acid stones and had better predictive value than BMI or urine pH in classifying the type of stone [12]. The main reason for the lack of a relationship between urine pH and stone composition may have been selection bias because of the diurnal variation of urine pH. The relationship between stone composition and MSD has been also determined by *in vitro* studies [13,14]. Classifying stones as urate, phosphate, and oxalate stones, different HU values have been found (urate: 513±197 HU; phosphate: 1660±292 HU; and oxalate: 1684±290 HU).

NCCT has become the gold standard for diagnosing urolithiasis [7]. NCCT can provide an abundance of information, including the size, number, and location of stones, and the presence of hydronephrosis [15]. However, there is now an interest in determining stone composition with NCCT, as stone composition can affect clinical decision-making and the outcomes of several therapeutic procedures. Dual-energy computed tomography (DECT) is a promising new technology that has the potential to improve our current ability to determine stone composition [16]. DECT was demonstrated to be effective for predicting uric acid and CaOx compound stones [17]. However, DECT has not become popular, and it has a higher risk of radiation hazards than low-dose or ultra-low-dose NCCT. In some countries, the price of DECT is higher than that of single-energy NCCT.

During the last decade, MSD has been a widely used NCCT parameter for characterizing urinary stones for both research purposes and clinical practice [18]. MSD can be a factor related to stone hardness; however, it is only an arithmetical average that cannot represent the heterogeneity of stone composition [9]. SHI has been defined as the standard deviation of the HU in a region of interest on NCCT [15]. The standard deviation of a random variable, statistical population, data set, or probability distribution is the square root of its variance. SHI is an index presenting the radiological heterogeneity of a urinary stone. Thus, SHI can represent the internal diversity of a stone, reflecting not only the heterogeneity of the stone’s composition but also the stone’s structural and morphological heterogeneity [9]. In a previous study of SHI by Lee et al., patients with uric acid stones may not have been included because the patients were selected on the basis of having radio-opaque ureter stones that could be treated easily by ESWL. By contrast, in the present study, we performed surgical intervention and extracted the stones, regardless of their x-ray opacity. This allowed us to demonstrate in the current study that SHI can be more powerful than MSD in predicting uric acid stones. Therefore, in uric acids stones, SHI can have two meanings in NCCT. First, since uric acid stone has low MSD and low SHI, SHI can serve as a predictor of uric acid stone. Another is that the formation of CaOx compound 100% stone in the density of NCCT is not homogeneous.

The European Association of Urology guidelines recommended that stone composition analysis be performed in all first-time stone formers [19] and conventionally, stone composition has been undoubtedly important in determining the efficacy of stone treatments, especially surgical interventions. The most significant differences have been found between radiolucent uric acid calculi (easily fragmented with shock-wave and dusted by Holmium:YAG laser) and relatively radiolucent cysteine calculi (often refractory to shock wave) [20]. Again, accurate prediction of the composition of urinary calculi is essential for choosing the optimal decision for treatment.

During the last 2 decades, the relationships between HU and stone composition have been investigated. Several studies demonstrated that stone composition could be predicted with high accuracy with an *in vitro* approach using HU and MSD [21–23]. Furthermore, Williams et al. suggested that knowing the major composition of a stone alone may not allow adequate prediction of its fragility during lithotripsy treatment, but variations in internal stone structure, including secondary mineral composition, may be a significant cause of variability of stone fragility [24]. Therefore, if both the internal structure and the composition of stone can be predicted, the combination may be the most significant predictor of fragility during lithotripsy treatment. Furthermore, Zarse et al. suggested that it is stone morphology, rather than X-ray attenuation, that correlates with fragility to shock waves in this common stone type [25]. Their report was very important regarding the microstructure of urinary stones; however, their focus was on CaOx compounds. Although not included in our study, cysteine stone has high MSD and low SHI, which may help with the discovery of cysteine stone in advance. In particular, if the presence of uric acid stones can be predicted, these stones can be expected to have better success rates with surgical intervention and possibly chemolitholysis or chemoprevention. Therefore, it will be possible to make more precise treatment decisions. In the current study, we have proposed a uric acid stone prediction model using MSD and SHI and reported corresponding cut-off values. Similar to the results of previous reports, uric acid stones were predictable when the MSD was < 572.3 HU. For SHI, our current results suggest that uric acid stones can be predicted when the SHI is < 140.4 HU. SHI has sensitivity and specificity were 0.855 (95% CI 0.794–0.904) and 0.829 (95% CI 0.679–0.928) for uric acid stones. This can be also confirmed by the ROC curve, and it can be confirmed that SHI is a more efficient factor in predicting uric acid stone than MSD.

This study has some inherent limitations because of its retrospective design, which may have introduced sampling bias. However, we were able to assemble a relatively large cohort of patients with various types of stone composition. This was possible, in part, because of the expanded indications for flexible ureteroscopic lithotripsy or retrograde intra-renal surgeries. Another limitation is that the actual proportion of uric acid stones was higher than the known incidence of approximately 10% in the general population. This may be because patients who were transferred to our tertiary hospital were less likely to be treated successfully with ESWL or medical expulsive therapy using tamsulosin, which may have increased the likelihood that they had uric acid stones. However, our relatively high percentage of uric acid stones allowed us to gain convincing evidence of the usefulness of SHI as NCCT parameters for predicting these types of stones. Before analyzing the results of this study, we believed that MSD and SHI did not affect our treatment choices. With our results, we have been influencing the choice of treatment and believe it should be presented as an option for treatment in the future. Also, additional research is needed to propose a model that predicts more accurate cut-off values and successful outcomes. To this day, MSL has been considered as the most powerful factor for success rate. The second powerful factor is MSD. In the same MSL and MSD stones, SHI can influence outcome success. However, in PCNL and URSL, surgeon’s skill can be a major factor for achieving stone-free status. Therefore, influences by these factors should be analyzed in a large-scale study.

## Conclusions

To our knowledge, this is the first study to report the relationship between stone composition and SHI. We proposed that SHI can be a useful new parameter to provide additional information to help discriminate stone composition. Our data indicate that SHI can predict uric acid stones, with a cut-off value of 140.4 HU. Using SHI to predict uric acid stones will provide a good opportunity to achieve improved success rates through surgical intervention and possibly chemolitholysis and chemoprevention.
